# Validation and Application of the Chinese Integral Nursing Leadership Scale Among Clinical Nurses

**DOI:** 10.1155/jonm/3672064

**Published:** 2026-07-26

**Authors:** Jingjing Zhang, Wenyi Jiang, Xinyu He, Lingli Hou, Yixuan Dai, Nixi Luo, Ya Yang, Jihui Lin, Congxia Hu

**Affiliations:** ^1^ Department of Nursing, The Affiliated Hospital, Southwest Medical University, Luzhou 646000, China, swmu.edu.cn; ^2^ Department of Cardiovascular Disease, The Affiliated Traditional Chinese Medicine Hospital, Southwest Medical University, Luzhou 646000, Sichuan, China, scu.edu.cn; ^3^ Wound Healing Basic Research and Clinical Application Key Laboratory of LuZhou, School of Nursing, Southwest Medical University, Luzhou 646000, Sichuan, China, scu.edu.cn; ^4^ Department of Endocrinology and Metabolism, The Affiliated Hospital, Southwest Medical University, Luzhou 646000, Sichuan, China, scu.edu.cn

**Keywords:** cultural adaptation, integral nursing leadership, nursing management, reliability, validity

## Abstract

**Background:**

The leadership of nurse managers critically influences the development of nursing teams and advancement of healthcare institutions. However, no validated instrument exists to assess integral leadership among Chinese nurse managers. Recently, the Integral Nursing Leadership Scale (INLS) has been developed and applied.

**Aim:**

To translate the INLS into Chinese, validate the psychometric properties of the Chinese version (CV‐INLS), and explore clinical nurses’ evaluations of integral nursing leadership among nurse managers.

**Methods:**

The INLS was translated into Chinese according to Brislin guidelines. A convenience sample of 662 nurses completed the 30‐item CV‐INLS. The psychometric properties of CV‐INLS, including structural validity and reliability, were evaluated following COSMIN guidelines. The validated CV‐INLS was subsequently used to assess clinical nurses’ perceptions of integral nursing leadership among Chinese nurse managers (*n* = 1, 023).

**Results:**

The content validity (I‐CVI = 0.929–1.000, S‐CVI/AVE = 0.993) of the CV‐INLS was satisfactory. Structural validity supported the four‐factor model with an acceptable model fit in the confirmatory factor analysis (CFI = 0.973, TLI = 0.970, RMSEA = 0.069, SRMR = 0.050). Multigroup CFA supported measurement invariance across age and education groups (configural, metric, and scalar/threshold invariance). Construct validity was supported by evidence of convergent and discriminant validity. In addition, excellent reliability was shown with an ordinal *α* of 0.942, *ω* of 0.943, and an ICC of 0.987. In the validation sample, the clinical nurses’ evaluations of nursing managers indicated moderately high leadership levels, with an overall CV‐INLS total score of 118.04 ± 29.69. Perceived integral nursing leadership levels were associated with age (30–39 years), educational level (bachelor’s degree or higher), years of experience (10 years or more), and working in an ICU setting.

**Conclusions:**

The CV‐INLS showed strong psychometric properties and can be used to assess perceived integral nursing leadership among nurse managers. Perceived integral nursing leadership was moderately high among nurses in the surveyed tertiary hospitals.

**Implications for Nursing Management:**

The CV‐INLS may provide useful insights into perceived integral nursing leadership among nurse managers and inform leadership development in tertiary hospital settings.

## 1. Introduction

Nurses, who are integral to the healthcare system, play a pivotal role in promoting public health and in addressing the aging population challenge [1, 2]. Nurse managers, as leaders of nursing teams, play a key role in shaping and enhancing the effectiveness of these teams. Their leadership has a profound impact on the professional identity of nurses, sense of belonging, job satisfaction, teamwork, and the overall working environment [3–5]. Nurse managers with strong leadership skills are instrumental in fostering the growth of both nursing teams and healthcare institutions [6]. The Chinese government has issued the National Nursing Career Development Plan (2021–2025), which highlights the importance of promoting scientific, standardized, and refined nursing management practices [7]. This policy underscores the growing significance of nursing leadership, which is a critical and timely issue within the nursing profession.

Currently, research on nursing manager leadership explores various leadership styles, including transformational, empowering, emotionally intelligent, authentic, and transactional styles [8]. These leadership styles can improve the service quality of nursing teams, but each of them has its limitations. For instance, transformational leadership focuses on the interests and motivations of team members, which may sometimes result in neglecting the emotional needs of nursing staff [9]. Authentic leadership, which emphasizes the leader’s self‐awareness, may lead to insufficient attention to team dynamics and external challenges [10]. Transactional leadership emphasizes job performance and task completion, which may stifle creativity and innovation within the team [11]. No single leadership style is perfectly suited to all nursing practice settings. Therefore, there is a critical need for an integrated leadership framework which is able to effectively address the complexities and dynamics of the evolving healthcare environment [12].

There is no widely used instrument in nursing to measure integral leadership. Building on Wilber’s integral theory, Cacioppe and Albrecht developed the “Integral Leadership and Management Profile.” They argued that integral leadership was not simply a variant of any single leadership style. Instead, they conceptualized it as a multilayered practice that linked the inner beliefs and values, their observable behaviors and competencies, and the culture and structures of the organization of managers [13, 14]. Meanwhile, in management research, Shaikh developed an integrative leadership measurement that combined six leadership styles, including transformational style, authentic style, ethical style, servant style, spiritual style, and transactional style. He suggested that effective leadership was inherently multidimensional. He also suggested that leaders needed to mix and match different styles as situations change, rather than adhering to a single style [10].

In the nursing management field, Cho and Choi introduced Wilber’s four quadrants of the integral model (intraindividual, extra‐individual, intracollective, and extra‐collective) into nursing leadership study. They proposed the concept of “integral nursing leadership” and developed the Integral Nursing Leadership Scale (INLS) [12]. Integral nursing leadership described the influence of nurse managers across four levels. The individual internal level was involved in values, self‐awareness, and emotional regulation. The individual external level focused on concrete leadership behaviors and management skills. The collective internal level was related to team culture, interpersonal relationships, and a shared vision. The collective external level referred to organizational structures, institutional arrangements, and performance orientation. The INLS divided integral nursing leadership into four linked domains that were individual leadership qualities, individual performance, influencing organizational culture, and organizational excellence. Studies of nursing samples in South Korea and other countries showed a stable factor structure and adequate reliability [12, 15]. In contrast, most leadership instruments in China focused on isolated leadership styles or restricted behavioral domains, and no scale had been developed specifically to evaluate integral nursing leadership [11]. Therefore, the INLS can be adapted for use in China as a tool to assess perceived integral nursing leadership.

Thus, this study translated the INLS into Chinese, validated it within a Chinese nursing population, and assessed the integral leadership of nurse managers based on nursing staff perceptions. This study provides an assessment tool for perceived integral nursing leadership in China and may provide a reference for nursing leadership education and training.

## 2. Methods

### 2.1. Study Design

This study was conducted in two phases. In Phase 1, the INLS was translated into Chinese and adapted cross‐culturally. In addition, the psychometric properties of the Chinese version of the INLS (CV‐INLS) were examined. In Phase 2, the validated CV‐INLS was used to evaluate perceived integral nursing leadership among Chinese nurse managers. Its psychometric properties were evaluated according to the Consensus‐based Standards for the Selection of Health Measurement Instruments (COSMIN) guidelines. The cross‐sectional survey was conducted following the Strengthening the Reporting of Observational Studies in Epidemiology (STROBE) statement.

### 2.2. Phase 1: Translation, Cross‐Cultural Adaptation, and Psychometric Evaluation

#### 2.2.1. Translation and Cross‐Cultural Adaptation

The developer of the original English INLS was contacted via email to obtain permission to translate the scale into Chinese (Supporting Figure S1). Subsequently, the INLS was translated using a forward–backward translation procedure based on Brislin’s guidelines [16].

The procedure involved five steps. (1) Forward translation: Two bilingual translators—one with a Ph.D. in medicine and the other with a Ph.D. in English—independently translated the English version of the INLS into Chinese. (2) Integration of forward translations: A nursing professor with international academic experience reviewed and resolved discrepancies, yielding a preliminary CV‐INLS. (3) Back‐translation: Two medical professors with international academic experience independently back‐translated the preliminary CV‐INLS into English. (4) Integration of back‐translations: An internationally experienced medical professor collaborated with the research team to resolve discrepancies between the original scale and the back‐translations, producing the final back‐translated English version. (5) Consultation with the original authors: After reviewing the original authors’ feedback on the back‐translated scale, adjustments were made to finalize the CV‐INLS. Finally, conceptual equivalence and accuracy were ensured through a systematic comparison of the original, translated, and back‐translated INLS versions.

#### 2.2.2. Delphi Expert Consultation and Pilot Testing

Two rounds of Delphi expert consultation were conducted. Fourteen experts evaluated the semantic comprehensibility and content validity of the CV‐INLS. The inclusion criteria for the experts were as follows: (1) expertise in nursing management, nursing education, health policy, or evidence‐based medicine; (2) employment at tertiary hospitals or undergraduate teaching institutions; (3) for experts with a bachelor’s degree, having an associate senior professional title or above; and (4) for experts with a master’s degree, having an intermediate professional title or above and working in a nursing management role. Demographic data of the experts are presented in Supporting Table S1. The experts evaluated the content validity of the CV‐INLS using a four‐point Likert scale (1 = very irrelevant, 2 = not relevant, 3 = relevant, and 4 = very relevant) and provided qualitative suggestions for item refinement.

After completing the translation and cross‐cultural validation of the CV‐INLS, 30 nurses were selected using purposive sampling for the pilot test [17]. The participants evaluated the clarity of the items and instructions using a dichotomous response format (clear/unclear).

#### 2.2.3. Participants and Sample Size

A total of 674 clinical nurses were recruited via convenience sampling at a tertiary hospital in Luzhou, China, between August and September, 2024. The inclusion criteria were as follows: (a) having a valid nursing license and (b) currently working in a clinical nursing position. The exclusion criteria included providing uniform responses, logical inconsistencies, and completion times of less than 4 min. Ultimately, 662 valid responses were obtained. The required sample size was determined using Kendall’s method, which recommends 5–10 participants per item [18]. As the scale comprised 30 items and assuming a 10% rate of invalid responses, the minimum required sample size was 165. The final sample was randomly divided into 260 and 402 participants for the exploratory factor analysis (EFA) and confirmatory factor analysis (CFA), respectively [19].

#### 2.2.4. Descriptive Statistics of the CV‐INLS

The CV‐INLS comprises 30 items across four dimensions rated on a 6‐point Likert scale. Descriptive statistics were calculated for the item and dimension scores. In addition, floor and ceiling effects were examined. Skewness and kurtosis were assessed to evaluate the score distributions at both the item and dimension levels [20].

#### 2.2.5. Content Validity

Content validity was assessed by calculating the content validity indices (I‐CVI and S‐CVI/ average variance extracted [AVE]) at the item and scale levels based on two Delphi rounds. The items were revised and finalized by incorporating qualitative feedback from experts. An I‐CVI of ≥ 0.78 and an S‐CVI/AVE of ≥ 0.90 were considered indicative of good content validity [21].

#### 2.2.6. Structural Validity

The structural validity was evaluated using EFA and CFA. The EFA was conducted using principal axis factoring with oblimin rotation. Before conducting the EFA, the Kaiser–Meyer–Olkin (KMO) measure of sampling adequacy and Bartlett’s test of sphericity were used to assess the suitability of the data for factor analysis. Adequacy was indicated by a KMO value ≥ 0.70 and a significant Bartlett’s test (*p* < 0.05) [22]. Factor loadings were reported using a pattern matrix from the oblimin rotation. The number of factors was primarily determined using Horn’s parallel analysis, which was further supplemented by examination of the scree plot and theoretical interpretability. Item retention was based on factor loading magnitude, cross‐loading patterns, and theoretical consistency.

Given that the items were measured on an ordinal Likert scale, CFA was conducted using a polychoric correlation matrix and weighted least squares mean and variance‐adjusted (WLSMV) estimation. Model fit was evaluated using the following criteria: A chi‐square to degrees of freedom ratio (*χ* ^2^/*d* *f*) between 1.0 and 3.0 (with values closer to 1.0 indicating better fit), root mean square error of approximation (RMSEA) < 0.08, standardized root mean square residual (SRMR) ≤ 0.08, and comparative fit index (CFI) and Tucker–Lewis index (TLI) > 0.90 [23]. To determine the optimal measurement structure, congeneric model, tau‐equivalent, correlated residuals, and second‐order factor models were compared based on theoretical considerations and model diagnostics. Established fit indices (CFI, RMSEA, and SRMR) were used to evaluate model adequacy.

#### 2.2.7. Measurement Invariance Across Demographic Groups

The measurement invariance of the scale across major sociodemographic groups was examined. The final measurement model comprised a four‐factor correlation structure. Given that the items were rated on a Likert scale ranging from 1 to 6 and were treated as ordered categorical variables, WLSMV estimation was used. Based on the sample distribution, age was analyzed in two groups (20–29 years, *n* = 270; ≥ 30 years, *n* = 392), and education level was categorized as junior college (*n* = 304) versus bachelor’s degree or above (*n* = 358). The invariance was tested hierarchically, including configural invariance (same factor structure), metric invariance (equal factor loadings), scalar invariance (equal intercepts), and strict invariances (equal residual variances). Model fit was assessed using CFI, TLI, RMSEA, and SRMR. Furthermore, differences in fit indices between nested models (ΔCFI and ΔRMSEA) were examined to determine whether the invariance constraints led to a substantial deterioration in model fit. In the comparison of adjacent nested models, measurement invariance was considered established if changes in fit indices met the following criteria: ΔCFI ≤ 0.010 and ΔRMSEA ≤ 0.015, with ΔSRMR ≤ 0.030 at the metric level and ΔSRMR ≤ 0.010 at the scalar/threshold level, indicating acceptable equality constraints [24].

#### 2.2.8. Convergent and Discriminant Validity

Convergent validity was assessed by calculating the composite reliability (CR) and AVE based on the final CFA model. Discriminant validity was examined by comparing the square root of the AVE for each factor with the interfactor correlation coefficients. AVE ≥ 0.50 and CR ≥ 0.70 were considered indicative of adequate convergent validity [25].

#### 2.2.9. Reliability

Internal consistency was assessed using ordinal *α* and ordinal *θ*, with McDonald’s omega (*ω*) used as a robustness check [26–28]. To evaluate the test–retest reliability, 30 clinical nurses were selected for reassessment using the CV‐INLS after a 2‐week interval. Scale stability was evaluated using the intraclass correlation coefficient (ICC) and a 95% confidence interval (CI). An ICC > 0.70 indicated satisfactory temporal stability [29].

### 2.3. Phase 2: Cross‐Sectional Survey: Evaluation of Nurse Managers’ Integral Leadership in China

#### 2.3.1. Participants and Sample Size

Between October 2024 and January 2025, clinical nurses were recruited from 22 provinces across China using convenience sampling. The geographic region was derived from the reported province and categorized as Eastern, Central, Western, or Northeastern China. The same inclusion criteria as in Phase 1 were applied. An additional 20% was added to the estimated sample size to account for any invalid questionnaires [18]. The survey consisted of 30 items; thus, a sample size of 170–360 participants was considered appropriate [30]. To enhance analytical robustness, 1112 questionnaires were distributed, yielding a total of 1023 valid responses (response rate: 92.0%).

#### 2.3.2. Participant Demographic and Professional Characteristics

Data on age, gender, educational level, department, shift schedule, years of experience, and tenure in the current position were collected.

#### 2.3.3. CV‐INLS

The CV‐INLS comprises 30 items organized into four subscales: Personal Leadership Qualities (9 items), Personal Leadership Performance (9 items), Personal Influence on Organizational Culture (7 items), and Personal Influence on Organizational Excellence (5 items). Participants rated their agreement with their nurse managers on a 6‐point Likert scale ranging from 1 = strongly disagree to 6 = strongly agree. The CV‐INLS total score was calculated as the sum of all 30 items. The total score ranged from 30 to 180, with higher scores indicating stronger perceived integral nursing leadership.

### 2.4. Data Collection and Ethical Considerations

Data were collected using both online and offline methods. Before completing the questionnaire, participants were provided with information on the study purpose, questionnaire content, and informed consent. Questionnaire completion was considered implied consent, and all responses were kept confidential.

Paper‐based surveys were administered onsite following institutional approval and participant consent. All completed questionnaires were checked before data entry. The online survey was administered via Questionnaire Star, a widely used and reliable online survey platform in China. Data quality was ensured by restricting each participant to a single submission and setting a designated timeframe for completion. Ethical approval was granted by the Biomedical Ethics Committee of Southwest Medical University (approval no. SWMUIRBTX‐202406‐0019).

### 2.5. Statistical Analysis

Data were analyzed using IBM SPSS Statistics Version 26.0 and R Version 4.5.2. Continuous and categorical variables were described as mean ± standard deviation and frequencies (percentages), respectively. Psychometric validation analyses were conducted in R Version 4.5.2 using the lavaan, semTools, and psych packages. All statistical tests were two‐tailed, and the significance level was set at *p* < 0.05.

Univariate analysis was conducted to examine the differences in the perceived integral nursing leadership of nurse managers according to nurses’ demographic characteristics. When homogeneity of variance was satisfied, independent‐sample *t*‐tests or one‐way ANOVA were performed. When the homogeneity of variance was not satisfied, corresponding nonparametric tests were performed. Subsequently, variables that were statistically significant (*P* < 0.05) in the univariate analyses were entered into the multivariate analysis. A multiple linear regression model was constructed using the CV‐INLS total score as the dependent variable. The analysis reported unstandardized regression coefficients (B), standard errors (SEs), standardized coefficients (*β*), 95% CIs, and *p* values. Model performance was summarized using *R* ^2^, adjusted *R* ^2^, and an overall model test. Multicollinearity was assessed using tolerance and the variance inflation factor (VIF). Residual diagnostics were conducted to evaluate the regression assumptions.

## 3. Results

### 3.1. Phase 1: Translation, Cross‐Cultural Adaptation, and Psychometric Validation

#### 3.1.1. Cultural Adaptation of INLS and Delphi Expert Consultation

Fourteen experts completed two Delphi rounds. In Round 1, both the response and feedback rates were 100%. In Round 2, the response rate was 100%, and the feedback rate was 64.2%. The expert authority coefficient (Cr = 0.921; Supporting Table S2) indicated high expert credibility. Experts proposed refinements to item wording and item order, which were implemented following discussions between the translation committee and the research team. During pilot testing, all 30 clinical nurses confirmed the clarity and cultural appropriateness of the Chinese items. Consequently, no semantic modifications were required. The finalized CV‐INLS is presented in Supporting Table S3.

#### 3.1.2. Characterization of Participants

This phase included 662 participants. The majority of participants were female (90.5%), aged 20–29 years (40.8%), and held a bachelor’s degree (54.1%). Nearly half of participants (44.7%) worked in clinical nursing roles. The mean years of experience was 2.66 (SD = 1.43), and the mean tenure in current position was 2.45 (SD = 1.41) (Supporting Table S4).

#### 3.1.3. Descriptive Statistics of the CV‐INLS

Assessment of item performance was conducted based on data from 662 participants. Results revealed generally high scores across all items. The mean scores of items ranged from 3.99 to 4.18, with standard deviations between 1.43 and 1.56. Item skewness values ranged from −0.60 to −0.77 and kurtosis values ranged from −0.82 to −0.42, indicating a moderate negative skew and a relatively platykurtic distribution. This was consistent with a mild clustering toward the higher end of the scale. Floor effects were observed in 6.19%–9.82% of responses, while ceiling effects ranged from 12.99% to 17.82%. This indicates a certain concentration of responses at the maximum score, particularly for the item “Personal Influence on Organizational Culture” (Supporting Table S5). After aggregating scores at the dimension and total levels, endpoint clustering was reduced, and floor effects ranged from 1.06% to 2.87%, while ceiling effects ranged from 0.30% to 3.17%, both below 5% (Supporting Table S6). This indicated no significant endpoint truncation at the dimension and total score levels, suggesting an adequate overall measurement range of the scale.

#### 3.1.4. Content Validity

The content validity of the CV‐INLS was assessed by 14 experts. The I‐CVI ranged from 0.929 to 1.00, and the S‐CVI/Ave was 0.993, indicating excellent content validity (Supporting Table S7).

#### 3.1.5. EFA

The KMO value was 0.846, and Bartlett’s test of sphericity was significant (*χ* ^2^ = 5761.192, *p* < 0.001), indicating suitability of the data for factor analysis. EFA was performed using principal axis factoring with oblimin rotation. The number of factors to retain was determined based on eigenvalues, the scree plot, and parallel analysis. Results showed that the initial eigenvalues for the first four factors were all greater than 1 (11.718, 4.614, 3.457, and 1.680), and the scree plot exhibited an inflection point after the fourth factor (Supporting Figure S2). Furthermore, Horn’s parallel analysis based on polychoric correlations and the 95th percentile criterion for random eigenvalues also supported the retention of four factors. The four factors cumulatively explained 66.97% of the total variance. The pattern matrix showed that item loadings on their respective factors ranged from 0.68 to 0.89 (Supporting Table S8). The absolute values of interfactor correlations ranged from 0.212 to 0.548 (Supporting Table S9), indicating that the dimensions were correlated but not overlapping. This supported the use of oblique rotation.

#### 3.1.6. CFA

**FIGURE 1:**
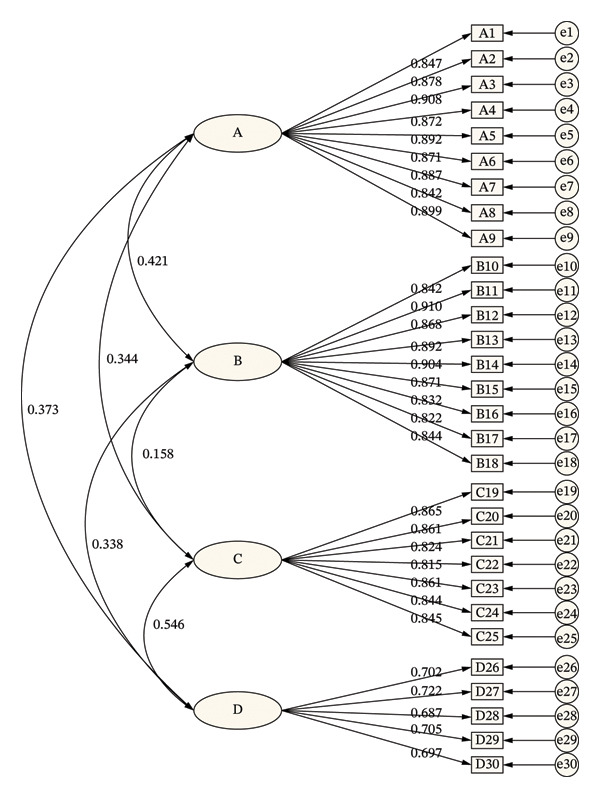
Four‐factor model. (A) Personal Leadership Qualities. (B) Personal Leadership Performance. (C) Personal Influence on Organizational Culture. (D) Personal Influence on Organizational Excellence.

CFA was conducted using the WLSMV estimator, supporting a four‐factor model of the CV‐INLS (Figure 1). The four‐factor correlated model demonstrated satisfactory fit: *χ* ^2^ (399) = 1152.39, *p* < 0.001, RMSEA = 0.069, 90% CI: [0.064, 0.073], SRMR = 0.050, CFI = 0.973, and TLI = 0.970. Under this model, the standardized factor loadings ranged from 0.687 to 0.910 across all items. Based on the results of the model comparison (Table 1), the unifactorial model showed poor fit and did not support a unidimensional structure. Incorporating residual correlations within the four‐factor framework resulted in only a marginal improvement (ΔCFI = 0.002), indicating no substantial enhancement. Both the tau‐equivalent model and the second‐order factor model showed only a minimal decrement in fit compared to the four‐factor correlated model (ΔCFI = −0.006 and −0.008, respectively). Therefore, the four‐factor correlated model was selected as the final measurement model.

**TABLE 1:** Fit indices for different CFA models.

Model	CFI	TLI	RMSEA	SRMR	ΔCFI
Congeneric model	0.705	0.684	0.224	0.247	−0.268
Four‐factor correlated model	0.973	0.970	0.069	0.050	0
Correlated residuals model	0.975	0.972	0.066	0.049	0.002
Tau‐equivalent model	0.967	0.967	0.073	0.052	−0.006
Second‐order factor model	0.965	0.962	0.078	0.071	−0.008

*Note:* ΔCFI is calculated with reference to the four‐factor correlation model.

Abbreviations: CFI, comparative fit index; RMSEA, root mean square error approximation; SRMR, standardized root mean square residual; TLI, Tucker–Lewis index.

#### 3.1.7. Measurement Invariance Across Demographic Groups

The results of the measurement invariance analysis are shown in Table 2. For the two educational groups (associate degree, *n* = 304; bachelor’s degree or above, *n* = 358), the correlated four‐factor configural model demonstrated good fit (CFI = 0.9986, RMSEA = 0.0265). Under the metric and scalar/threshold constraints, changes in fit indices were small (metric: ΔCFI = −0.0009, ΔRMSEA = 0.0065, ΔSRMR = 0.0027; scalar/threshold: ΔCFI = 0.0012, ΔRMSEA = −0.0116, ΔSRMR = −0.0027), and all values were below the prespecified cutoffs. These results supported measurement invariance across education groups.

**TABLE 2:** The measurement invariance of the CV‐INLS (education level and age).

Model	*χ* ^2^ (df)	*χ* ^2^/df	TLI	CFI	RMSEA	SRMR	ΔCFI	ΔRMSEA	ΔSRMR
*Panel A: Educational level (junior college vs. bachelor’s degree or above)*									
Configural invariance	1427.998 (798)	1.7895	0.9985	0.9986	0.0265	0.0395			
Metric invariance	1391.169 (824)	1.6883	0.9976	0.9977	0.033	0.0423	−0.0009	0.0065	0.0027
Scalar invariance	1492.967 (914)	1.6334	0.999	0.9989	0.0214	0.0396	0.0012	−0.0116	−0.0027
Residual invariance	1492.085 (944)	1.5806	0.9983	0.9982	0.0275	0.0423	−0.0007	0.0061	0.0027

*Panel B: age (20∼29 vs. ≥ 30)*									
Configural invariance	1447.817 (798)	1.8143	0.9981	0.9983	0.0292	0.0402			
Metric invariance	1436.512 (824)	1.7433	0.997	0.9971	0.0369	0.0433	−0.0011	0.0077	0.0031
Scalar invariance	1539.837 (914)	1.6847	0.9985	0.9984	0.0261	0.0403	0.0013	−0.0108	−0.003
Residual invariance	1591.137 (944)	1.6855	0.9973	0.9971	0.0349	0.0434	−0.0013	0.0088	0.0031

*Note:* *χ* ^2^, chi‐square.

Abbreviations: CFI, comparative fit index; df, degrees of freedom; RMSEA, root mean square error approximation; SRMR, standardized root mean square residual; TLI, Tucker–Lewis index.

For the two age groups (20–29 years, *n* = 270; ≥ 30 years, *n* = 392), the correlated four‐factor configural model showed good fit (CFI = 0.9983, RMSEA = 0.0292). Under the metric and scalar/threshold constraints, changes in fit indices were small (metric: ΔCFI = −0.0011, ΔRMSEA = 0.0077, ΔSRMR = 0.0031; scalar/threshold: ΔCFI = 0.0013, ΔRMSEA = −0.0108, ΔSRMR = −0.0030), and all values were below the prespecified cutoffs. These results supported measurement invariance across age groups (Table 2).

#### 3.1.8. Convergent and Discriminant Validity

The convergent and discriminant validity of the CV‐INLS is presented in Supporting Table S10. The CR values for all four dimensions exceeded 0.70, and the AVE values ranged from 0.494 to 0.771. Notably, Dimension 4 exhibited an AVE value of 0.494, slightly below the threshold of 0.50. According to Fornell and Larcker, when AVE is below this threshold, but CR exceeds 0.60, convergent validity can still be considered adequate [31]. In this study, the CR was 0.830, indicating acceptable convergent validity for this dimension. In terms of discriminant validity, the square root of the AVE for each dimension was greater than its correlations with other dimensions, providing evidence of discriminant validity.

#### 3.1.9. Reliability

As shown in Table 3, the ordinal *α* for the total scale was 0.942, *θ* was 0.944, and *ω* was 0.943, indicating high overall internal consistency. Test–retest reliability assessed in 30 nurses after a 2‐week interval demonstrated strong temporal stability (ICC = 0.987 for the total scale, 0.790–0.917 across subscales). Therefore, the CV‐INLS demonstrated excellent internal consistency and temporal stability.

**TABLE 3:** Result of reliability analysis of the CV‐INLS (*n* = 662).

Dimension	No.	Ordinal *α*	*θ*	ꞷ	ICC
1. Personal Leadership Qualities	9	0.967	0.967	0.967	0.826
2. Personal Leadership Performance	9	0.963	0.963	0.963	0.790
3. Personal Influence on Organizational Culture	7	0.945	0.945	0.945	0.917
4. Personal Influence on Organizational Excellence	5	0.831	0.831	0.831	0.891
Total scale	30	0.942	0.944	0.943	0.987

*Note: α*, Cronbach’s alpha; ꞷ, McDonald’s omega.

Abbreviation: ICC, intraclass correlation coefficient.

To sum up, the CV‐INLS can be used as a valid and reliable instrument for assessing evaluations of clinical nurses on integral leadership of nurse managers in China.

### 3.2. Phase 2: Perceptions and Evaluation of Clinical Nurses on Integral Leadership of Nurse Managers in China

#### 3.2.1. Participant Characteristics

This phase included 1, 023 clinical nurses. Based on regional distribution, samples from the eastern region accounted for 59.6%, while those from the central, western, and northeastern regions accounted for 12.31%, 15.61%, and 12.46%, respectively. Participants were predominantly female (90.91%), aged 20–29 years (43.59%), and held a bachelor’s degree or higher (56.89%). Most participants worked in medical (43.11%) or surgical (37.15%) units, and 53.76% worked rotating shifts. Years of experience were primarily 1–9 (49.17%) or 10–19 (33.33%), and 51.71% had 1–9 years of tenure in their current position (Table 4).

**TABLE 4:** Demographic characteristics of participating nurses (*n* = 1, 023).

Variable	*n*	%
Sample source region		
Eastern	610	59.6
Central	126	12.31
Western	160	15.61
Northeastern	127	12.46
Age (years)		
20∼29	446	43.59
30∼39	335	32.75
≥ 40	242	23.66
Gender		
Male	93	9.09
Female	930	90.91
Educational level		
Junior college	441	43.11
Bachelor’s degree or above	582	56.89
Department		
Medical	441	43.11
Surgical	380	37.15
Intensive care unit (ICU)	83	8.11
Others^∗^	119	11.63
Shift schedule		
Day–evening–night	550	53.76
Day–evening	247	24.14
Fixed night	131	12.81
Fixed day (9 am–5 pm)	95	9.29
Experience (years)		
1∼9	503	49.17
10∼19	341	33.33
≥ 20	179	17.50
Tenure in the current position		
1∼9	529	51.71
9∼16	262	25.61
≥ 16	232	22.68

*Note:* The regions are categorized based on provincial information according to the national statistical zoning system.

^∗^Others include gynecology, obstetrics, emergency, and operating room.

#### 3.2.2. Level of Perceived Integral Nursing Leadership

The CV‐INLS total score was 118.04 ± 29.69, indicating a moderately high level of leadership among Chinese nurse managers as perceived by clinical nurses. The scores for the four dimensions were 36.41 ± 11.44 for Personal Leadership Performance, 36.35 ± 11.41 for Personal Leadership Qualities, 28.84 ± 8.77 for Personal Influence on Organizational Culture, and 24.62 ± 7.12 for Personal Influence on Organizational Excellence. The detailed item‐level scores are shown in Supporting Table S11.

#### 3.2.3. Differences in Perceived Integral Nursing Leadership by Participant Characteristics

Univariate analyses revealed significant differences in the overall CV‐INLS total score among nurses with different ages, genders, educational levels, department types, years of experience, and tenure in the current position (*p* < 0.05). The shift schedule was not significantly associated with perceived integral nursing leadership (*p* = 0.792) (Supporting Table S12).

#### 3.2.4. Factors Associated With Perceived Integral Nursing Leadership

To further identify factors associated with perceived integral nursing leadership, the CV‐INLS total score was used as the dependent variable. Variables that were statistically significant in univariate analysis (age, gender, educational level, department type, years of experience, and tenure in the current position) were entered into a multiple linear regression model (Table 5). The model was statistically significant (*F* = 81.88, *p* < 0.001) and accounted for 36.1% of the variance. Factors positively associated with the CV‐INLS total score included bachelor’s degree or above (*β* = 0.147, *p* < 0.001), older age (*β* = 0.197, *p* = 0.003), and longer work experience (*β* = 0.186, *p* = 0.010). Conversely, working in the ICU was negatively associated with the CV‐INLS total score (*B* = −5.881, *β* = −0.194, *p* < 0.001).

**TABLE 5:** Multiple linear regression analysis of factors associated with the CV‐INLS total score.

Variables	*B*	SE	*β*	*t*	*P*
Age (30∼39)	7.351	2.456	0.197	2.993	0.003
Education (bachelor’s degree or above)	8.821	1.524	0.147	5.789	< 0.001
Department (ICU)	−5.881	0.770	−0.194	−7.638	< 0.001
Years of experience	7.351	2.862	0.186	2.569	0.01

*Note:* Dependent variable: CV‐INLS total score (sum of 30 items, range: 30–180); *B* = unstandardized regression coefficients; *β* = standardized coefficients; *t* = B/SE.

Abbreviation: SE = standard error.

## 4. Discussion

This study accomplished the translation, cross‐cultural adaptation, and psychometric validation of the INLS [16]. The findings support the assessment of perceived integral nursing leadership in similar Chinese clinical settings.

The healthcare system in China has become more complex following the COVID‐19 pandemic, alongside rising demand driven by population aging and chronic diseases [32–34]. Therefore, improving the quality of nursing services is a national priority [7]. Nurse managers are required to make timely decisions while supporting staff wellbeing and morale [35–37]. Many existing leadership tools focus on single dimensions or styles and may be unable to capture these combined demands [12, 38]. This underscores the need for an integral leadership approach and measurement instrument aligned with current practice [39]. To support the practical application of CV‐INLS, the Chinese items were required to retain their original meaning while remaining clear and appropriate for Chinese nurses.

Fourteen multidisciplinary experts participated in the cultural adaptation process. Their expertise and familiarity with the Chinese healthcare context ensured that the adapted items remained conceptually consistent with the original version. Consultations with respondents during pilot testing further supported the clarity, comprehensibility, and contextual appropriateness of the items. Moreover, both I‐CVI and S‐CVI/Ave exceeded the recommended thresholds (I‐CVI > 0.78 and S‐CVI/Ave > 0.90) [21], suggesting a consensus regarding the relevance and representativeness of the items among experts.

The overall item‐level scores were high, suggesting a potential ceiling effect, with approximately 12.99%–17.82% of responses given the maximum score. This phenomenon is common in leadership‐related scales and may be attributed to social desirability or courtesy bias [40]. In addition, the sample predominantly comprised younger female nurses in clinical roles. Their potential reliance on general impressions during scoring may have contributed to a mildly negative skew in item distribution. After aggregation at the dimensional and total score levels, floor and ceiling effects were substantially reduced to below the commonly cited threshold of 15% [41]. This indicates that the scale retained an adequate measurement range at these broader levels, thereby reducing the potential impact of endpoint truncation on comparative and longitudinal analyses.

Structural validity supported the four‐dimensional correlated model. Both EFA and CFA revealed this pattern. The factor correlations were moderate. This aligns with the theoretical expectation that integral nursing leadership is multidimensional, interrelated, and distinguishable. Four factors were extracted via EFA, accounting for 66.97% of the cumulative variance. Factor loadings indicated that the items were generally aligned with their respective dimensions. This structure was aligned with the original four‐dimensional model and confirmed the findings from the Persian‐language validation study [12, 42]. It is worth noting that the cumulative explained variance in the Persian validation study was higher (approximately 81.58%). This discrepancy may stem partly from the methodological choices and sample differences. Specifically, principal component analysis tends to yield a higher explained variance than common‐factor methods [43]. The principal axis factoring method employed in this study was more closely aligned with the latent variable measurement objectives. Therefore, the explained variance should not be interpreted simply as a higher value being better. Instead, its evaluation may require considering the extraction method and theoretical coherence.

In the CFA phase, WLSMV estimation was used, and the items were treated as ordinal variables, consistent with the recommended modeling practices for Likert ordinal responses. This approach reduced the parameter estimation and model‐fit bias when ordinal data were treated as continuous [44]. The four‐factor model achieved commonly accepted levels of fit, whereas the unidimensional model demonstrated poor fit. This supported the conclusion that the scale measured not only a single trait but also a composite of related yet distinguishable dimensions. Compared to the fit levels reported for the Persian version of the scale (RMSEA ≈ 0.063, CFI ≈ 0.98, SRMR ≈ 0.043), the fit indices in this study fell within a similar range, suggesting that the four‐factor structure possessed a degree of cross‐cultural stability [42]. Furthermore, the model comparisons in this study included correlated residuals, equal loadings, and second‐order models. These comparisons showed not only limited improvement in model fit but also a clearer theoretical interpretability for the four‐factor correlated model. Therefore, the four‐factor correlation model was retained. This could facilitate subsequent dimension‐based interpretation and application as well as avoid excessive reliance on data‐driven model modifications.

The measurement invariance testing provided critical incremental evidence for this study. The analyses supported scalar/threshold invariance across educational levels and age groups, indicating that the groups interpreted the items similarly. These findings provide a psychometric basis for group comparisons, such as differences in leadership perceptions across educational backgrounds or age cohorts. This is particularly important for nursing management research. Without evidence of invariance, the observed group differences may have been confounded by measurement bias. The criteria were based on the thresholds for ΔCFI, ΔRMSEA, and ΔSRMR, which were consistent with widely cited recommendations in structural equation modeling [45]. Compared to the original development study and the Persian validation study, which primarily focused on structural validity and internal consistency, the current study provides additional evidence for cross‐group comparability. Consequently, the CV‐INLS may be useful in studies involving educational or age stratification as well as in organizational‐level assessment and improvement‐related contexts.

The convergent and discriminant validity results further supported the interpretation of the dimensional structure. The CR values for all dimensions exceeded 0.70, and the AVEs were within the acceptable range. Moreover, the AVE square root for each dimension surpassed the interdimensional correlations, which aligns with the Fornell–Larcker discriminant validity testing approach [20]. Dimension 4 exhibited an AVE slightly below 0.50, yet its CR and loadings remained high, indicating that although this dimension showed a relatively weaker variance explanation, it still formed a consistent measurement set.

Regarding reliability, ordinal *α*, *θ*, and *ω* were reported, and a 2‐week test–retest ICC was provided. Taken together, these indices provide evidence of internal consistency and temporal stability. For Likert‐type ordinal ratings, ordinal reliability coefficients based on polychoric correlations were better aligned with the data characteristics than Cronbach’s *α* [27]. In this study, the ordinal reliability coefficient for the total scale was 0.942, indicating good internal consistency. The test–retest ICC was 0.987, indicating strong short‐term score stability. This finding is consistent with those of previous psychometric assessments of leadership and management scales in healthcare contexts [25, 46]. Compared to the original INLS (*α* ≈ 0.97, *ω* ≈ 0.98) and the Persian version (*α* ≈ 0.935, *ω* ≈ 0.949), the CV‐INLS demonstrated similar reliability, thereby indicating that language conversion did not result in significant reliability loss.

Therefore, the CV‐INLS can be used to assess perceived integral nursing leadership among Chinese nurse managers in similar tertiary hospital settings. Subsequently, using the validated CV‐INLS, we assessed nurse managers’ leadership from the perspective of 1023 clinical nurses. The results indicated moderately high levels of perceived integral nursing leadership, suggesting room for further development.

Both univariate analyses and multiple linear regression indicated that higher integral leadership ratings of nurse managers were associated with older age, higher educational level, and greater years of experience. In the regression model, age and years of experience were significantly positively associated with integral leadership ratings. Previous studies suggested that leadership capabilities develop over time through the accumulation of clinical experience [6]. Higher educational preparation was also associated with stronger decision‐making, clearer communication, and greater readiness to lead change [47]. These characteristics may be associated with easier recognition of effective leadership behaviors while rating managerial performance. However, there was considerable variation in the total score even within the same age or seniority group. This indicates that seniority alone does not reflect perceived leadership capabilities. When the years of service and educational background are considered together, the scale may provide additional information for leadership assessments.

Notably, after adjusting for other variables, ICU work was found to be negatively associated with leadership ratings. However, this does not necessarily mean that ICU nurse managers had poorer leadership skills. Instead, it may reflect higher expectations in the ICU setting. In high‐risk, high‐workload units, nurse managers may be expected to demonstrate integral leadership aligned across individual qualities, team culture, and organizational support. Previous studies have shown that ICU nurses perform intensive monitoring and frequent resuscitation work [48], making them especially sensitive to staffing levels, fair rostering, and timely decision support. If these resources or supports are inadequate, integral leadership may be rated less favorably. ICU managers often use a directive‐style approach to ensure patient safety and efficiency during emergencies. When tasks dominate, they may neglect to build a team culture, support personal growth, and care for the emotional needs of nurses [11].

Previous studies have indicated that discrepancies exist between the self‐evaluations of leaders and the assessments of staff, in which employee perceptions could directly influence behavioral outcomes [49, 50]. As an observer‐assessment tool, the CV‐INLS could provide useful feedback for leadership development, training, and management improvement initiatives. First, it could help clarify the leadership competencies of clinical nurse managers, identify dimensions where nurse managers are consistently recognized by staff, and recognize areas that may require further development. However, it is not intended to be used on a standalone basis for promotion or demotion. In addition, the results could be presented in a stratified manner, such as by department or years of experience, to support a more balanced interpretation of the staff ratings across different groups. Second, the four dimensions of the scale can be used to inform training programs by translating them into specific management competency modules. The Personal Leadership Qualities dimension focuses on self‐management and equity. This domain includes self‐awareness and accountability, emphasizing respect for staff and fair problem‐solving. The Personal Leadership Performance dimension, which includes interpreting policies, managing rosters, and using digital tools in routine management, focuses on managers’ day‐to‐day operational skills. The Personal Influence on Organizational Culture dimension focuses on team atmosphere and psychological safety. It aims to create an environment wherein staff can speak up, work well together, and constructively address conflict. It also embeds patient safety standards into daily practice. The Personal Influence on Organizational Excellence dimension focuses on strategic alignment and continuous improvements. For instance, managers can use small, feasible quality improvement projects (e.g. catheter care or fall prevention) to translate policies into clear processes and routines. Subsequently, data tracking can be used to monitor outcomes and support sustained improvements. Third, the CV‐INLS can be used as a feedback tool for leadership development. Periodic assessments based on clinical nurses’ evaluations of nursing managers’ integral leadership could enable hospital administrators to identify potential areas for improvement and inform leadership training programmes.

Overall, perceived integral nursing leadership is an evolving process that supports the development of leaders, teams, and organizational outcomes [12]. Compared to instruments that focus on a single leadership style or type, the CV‐INLS assesses perceived integral nursing leadership across multiple dimensions. Although its application should be approached with caution owing to the current sample’s specific characteristics, the scale could provide a useful reference for leadership assessment, training, and professional development in nursing management.

## 5. Limitations

This study has certain limitations. Measurement invariance was tested across several sociodemographic groups. However, invariance was not tested by sex because the sample was highly unbalanced. This imbalance might reduce the stability of multigroup estimates. In this study, the sample was selected through convenience sampling, which might introduce sampling bias. Most participants (59.6%) were from the eastern region, where healthcare resources were generally better. This concentration in more developed areas might have contributed to the relatively high CV‐INLS total score. Participants were exclusively recruited from tertiary hospitals. This recruitment limited the generalizability of the findings to nurses in primary or secondary hospitals, community healthcare centers, or mental health institutions. Due to the cross‐sectional design in Phase 2, causal relationships could not be inferred. Future longitudinal studies would be needed to establish causality. In addition, future studies should consider expanding sample diversity by including participants from different hospital levels, geographical regions, and healthcare settings. They should also include a more balanced sex distribution to support invariance testing by sex and to further validate the applicability of the CV‐INLS.

## 6. Conclusion

The CV‐INLS and its four subscales possess excellent internal consistency, test–retest reliability, and satisfactory structural validity. Thus, the CV‐INLS can serve as a reliable instrument for assessing perceived integral nursing leadership among nurse managers. In addition, this study surveyed the perceptions of clinical nurses about the leadership competencies of their managers, revealing a moderately high level of perceived integral nursing leadership. Perceived integral nursing leadership levels were associated with age (30–39 years), educational level (bachelor’s degree or higher), years of experience (10 years or more), and working in an ICU setting. These findings suggest that further development of perceived integral leadership capabilities among nurse managers in similar contexts may be beneficial.

## 7. Implications for Nursing Management

The CV‐INLS can serve as a useful tool for assessing perceived integral nursing leadership among nurse managers. It can provide useful information for leadership education, training, and professional development assessment for nurse managers in similar tertiary hospital settings. The scale may also support the evaluation of leadership development initiatives based on nurse perceptions and inform evidence‐informed nursing management practices within these contexts.

## Author Contributions

Jingjing Zhang: methodology, data curation, formal analysis, investigation, writing–original draft, and writing–review and editing. Wenyi Jiang: conceptualization, methodology, formal analysis, investigation, writing–original draft, and writing–review and editing. Xinyu He: conceptualization, formal analysis, investigation, and writing–review and editing. Lingli Hou: data curation, formal analysis, and investigation. Nixi Luo: formal analysis, investigation, and writing–review and editing. Yixuan Dai: conceptualization, data curation, and writing–original draft. Ya Yang: conceptualization, investigation, and data curation. Jihui Lin: conceptualization, methodology, validation, project administration, and writing–review and editing. Congxia Hu: conceptualization, methodology, validation, supervision, project administration, and writing–review and editing.

## Funding

This study was supported by the Sichuan Science and Technology Program (Grant no. 2025ZNSFSC0676), the Luzhou Science and Technology Program (Grant no. 2025JYJ070), the Scientific Research Startup Fund of the Affiliated Hospital of Southwest Medical University (Grant no. 25050), and the Southwest Medical University Technology Program (Grant no. 2025JC012).

## Disclosure

All authors reviewed the manuscript.

## Conflicts of Interest

The authors declare no conflicts of interest.

## Supporting Information

Additional supporting information can be found online in the Supporting Information section.

## Supporting information


**Supporting Information** Supporting Figure S1. Authorization from original scale developers. Supporting Figure S2. Scree plots of parallel analysis. Supporting Table S1. Demographic characteristics of Delphi experts (*n* = 14). Supporting Table S2. Calculation of expert authority coefficient. Supporting Table S3. Chinese version of the INLS (CV‐INLS). Supporting Table S4. Demographic characteristics of psychometric validation participants (*n* = 662). Supporting Table S5. Item‐level descriptive statistics of the CV‐INLS (*n* = 662). Supporting Table S6. Descriptive statistics of the CV‐INLS (*n* = 662). Supporting Table S7. Content validity of the CV‐INLS. Supporting Table S8. Factor loadings of the CV‐INLS items (*N* = 662). Supporting Table S9: Factor correlation matrix from EFA. Supporting Table S10. Results of the convergent and discriminant validity of the CV‐INLS. Supporting Table S11. Item and dimension mean scores of the CV‐INLS (*n* = 1023). Supporting Table S12. Differences in perceived integral nursing leadership among nurses with different characteristics (*n* = 1023).

## Data Availability

The datasets used and/or analyzed during the current study are available from the corresponding author upon reasonable request.
