# Supplementary material for: Validation and Application of the Chinese Integral Nursing Leadership Scale Among Clinical Nurses
Source: J Nurs Manag. 2026 Jul 26;2026:3672064. doi: 10.1155/jonm/3672064 (PMC13402858; doi:10.1155/jonm/3672064)
Supplement: Supplementary file 1 — Supporting Information Supporting Figure S1. Authorization from original scale developers. Supporting Figure S2. Scree plots of parallel analysis. Supporting Table S1. Demographic characteristics of Delphi experts (n = 14). Supporting Table S2. Calculation of expert authority coefficient. Supporting Table S3. Chinese version of the INLS (CV‐INLS). Supporting Table S4. Demographic characteristics of psychometric validation participants (n = 662). Supporting Table S5. Item‐level descriptive statistics of the CV‐INLS (n = 662). Supporting Table S6. Descriptive statistics of the CV‐INLS (n = 662). Supporting Table S7. Content validity of the CV‐INLS. Supporting Table S8. Factor loadings of the CV‐INLS items (N = 662). Supporting Table S9: Factor correlation matrix from EFA. Supporting Table S10. Results of the convergent and discriminant validity of the CV‐INLS. Supporting Table S11. Item and dimension mean scores of the CV‐INLS (n = 1023). Supporting Table S12. Differences in perceived integral nursing leadership among nurses with different characteristics (n = 1023). [file JONM-2026-3672064-s001.docx]

**Supplementary Figures and Tables**


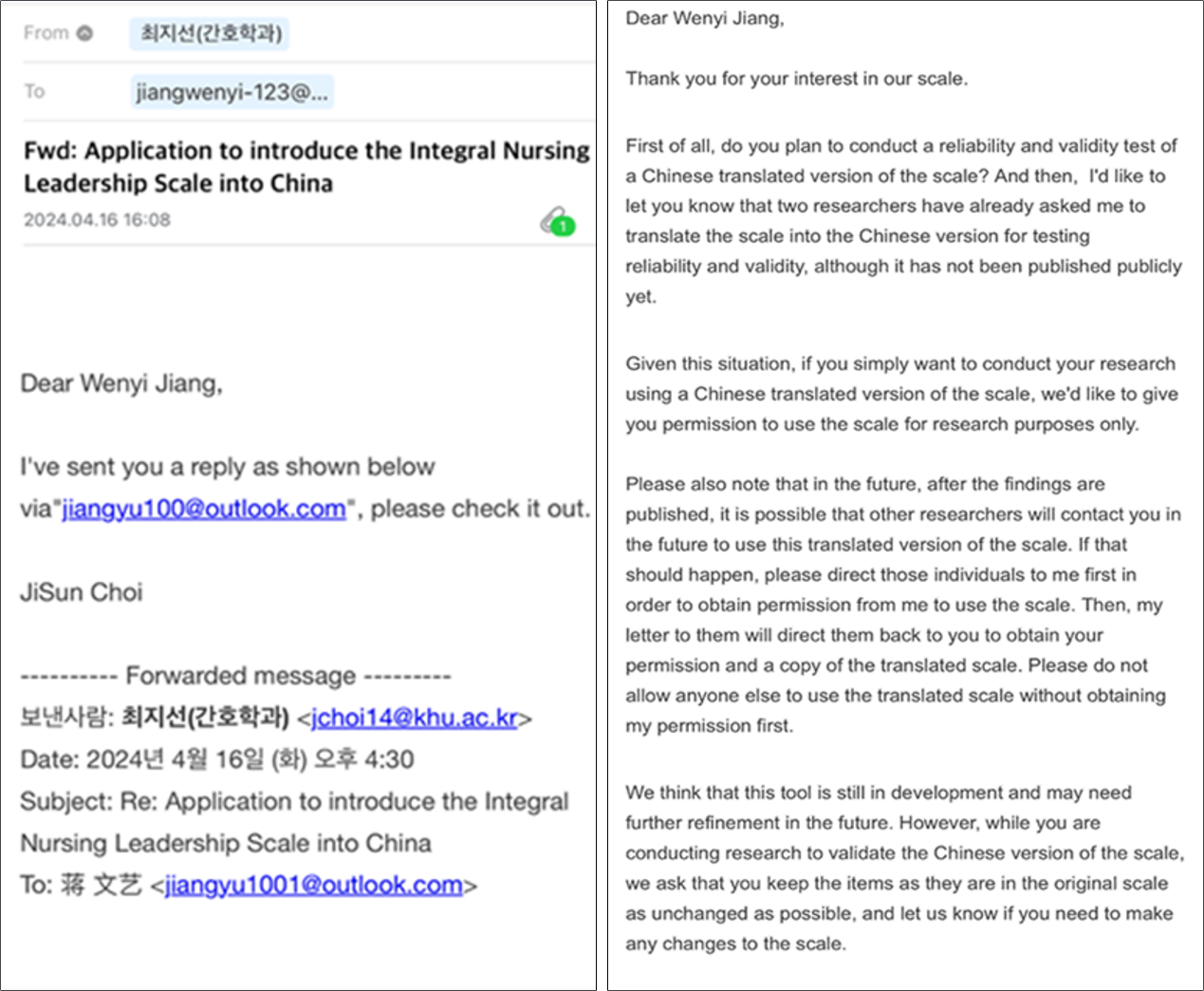


**Supplementary Figure S1.** Authorization from Original Scale Developers.


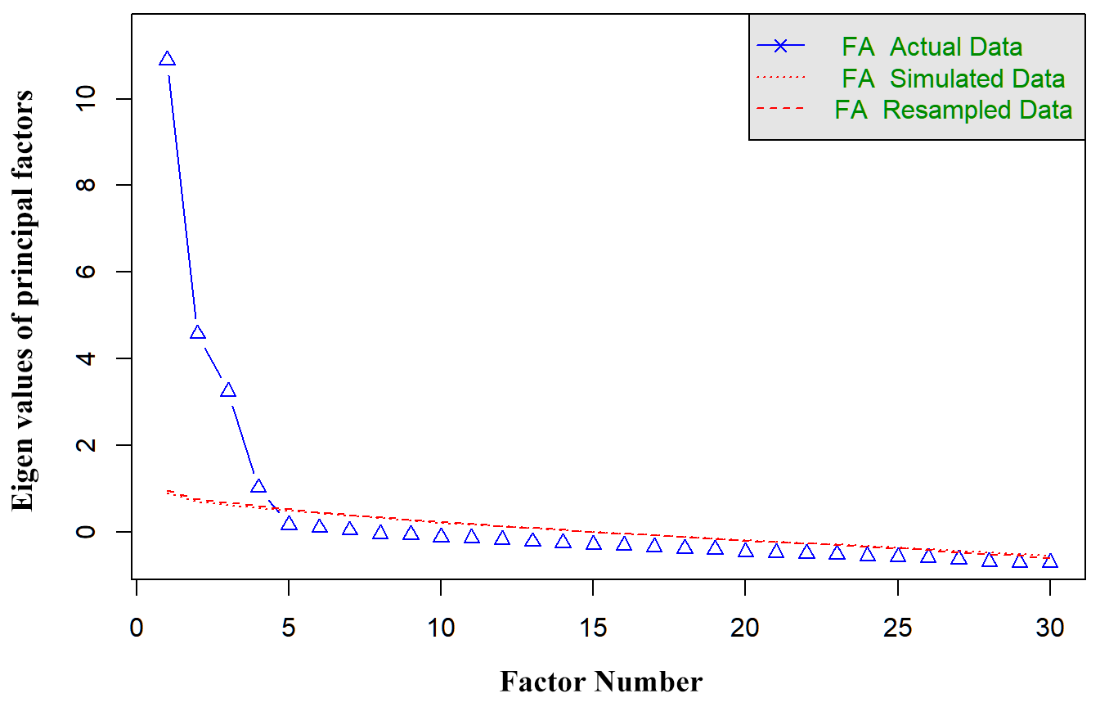


**Supplementary Figure S2.** Scree plots of parallel analysis

**Supplementary Table S1.** Demographic Characteristics of Delphi Experts (n = 14).

| **No.** | **Educational background** | **Professional title** | **Years of employment** | **Mentor** | **Research field** |
| --- | --- | --- | --- | --- | --- |
| 1 | Master’s | Professor | 25 | Yes | Healthcare Administration |
| 2 | Master’s | Chief Nurse | 41 | Yes | Clinical Nursing Management, Geriatric Nursing |
| 3 | PhD | Chief Nurse | 26 | Yes | Nursing Management, Chronic Disease Care, Nursing Education |
| 4 | PhD | Professor | 40 | Yes | Nursing Management, Nursing Education |
| 5 | PhD | Professor/Researcher | 5 | Yes | Evidence-Based Medicine |
| 6 | Master’s | Professor | 37 | Yes | Nursing Education, Nursing Management, Clinical Nursing |
| 7 | Master’s | Associate Chief Nurse | 21 | Yes | Clinical Nursing, Nursing Education |
| 8 | Bachelor’s | Professor | 41 | Yes | Nursing Education |
| 9 | Master’s | Associate Chief Nurse | 20 | Yes | Nursing Management |
| 10 | Master’s | Associate Professor | 29 | Yes | Clinical Nursing, Nursing Education |
| 11 | Master’s | Chief Nurse | 30 | Yes | Nursing Management, Hospital Administration |
| 12 | Master’s | Professor | 42 | Yes | Pediatric Pain Management, Chronic Pediatric Care, Nursing Education |
| 13 | PhD | Chief Nurse | 37 | Yes | Health Management in Cerebrovascular Patients |
| 14 | Master’s | Associate Chief Nurse | 35 | Yes | Nursing Management, Hospital Administration |

**Note:** PhD, Doctor of Philosophy.

**Supplementary Table S2.** Calculation of Expert Authority Coefficient.

| Round | The degree of familiarity of experts with the contents of the inquiry (Cs) | The basis on which experts judge the content of the inquiry (Ca) | Authority coefficient (Cr) |
| --- | --- | --- | --- |
| The first round | 0.900 | 0.942 | 0.921 |
| The second round | 0.889 | 0.921 | 0.904 |

**Supplementary Table S3.** Chinese version of the Integral Nursing Leadership Scale (CV-INLS).

| 1.你了解上级的优点和缺点  □非常不同意 □不同意 □不太同意 □部分同意  □同意 □非常同意 |
| --- |
| 2.你能够认识到上级作为领导者的角色和能力  □非常不同意 □不同意 □不太同意 □部分同意  □同意 □非常同意 |
| 3.你的上级能清楚表达自己的信念和价值观  □非常不同意 □不同意 □不太同意 □部分同意  □同意 □非常同意 |
| 4.你能评估上级作为领导者的表现  □非常不同意 □不同意 □不太同意 □部分同意  □同意 □非常同意 |
| 5.你能客观认识到上级的想法、情感和行为  □非常不同意 □不同意 □不太同意 □部分同意  □同意 □非常同意 |
| 6.你的上级能够尊重员工并维护员工的尊严  □非常不同意 □不同意 □不太同意 □部分同意  □同意 □非常同意 |
| 7.你的上级能够把员工视为独立个体，而不仅仅是组织的一员  □非常不同意 □不同意 □不太同意 □部分同意  □同意 □非常同意 |
| 8.你的上级能够履行领导职责、具有责任担当  □非常不同意 □不同意 □不太同意 □部分同意  □同意 □非常同意 |
| 9.你的上级会把组织的成就归功于员工个人或全体组织，而非她/他自己个人  □非常不同意 □不同意 □不太同意 □部分同意  □同意 □非常同意 |
| 10.你的上级会积极寻找创新方法来提高护理服务质量  □非常不同意 □不同意 □不太同意 □部分同意  □同意 □非常同意 |
| 11.你的上级能洞察到医疗环境和相关政策的变化  □非常不同意 □不同意 □不太同意 □部分同意  □同意 □非常同意 |
| 12.你的上级能分析和解读护理服务相关的数据  □非常不同意 □不同意 □不太同意 □部分同意  □同意 □非常同意 |
| 13.你的上级能够在护理工作（包括护理实践和护理教学）中使用数字技术  □非常不同意 □不同意 □不太同意 □部分同意  □同意 □非常同意 |
| 14.你的上级能有效制定并管理护理单元的运营成本  □非常不同意 □不同意 □不太同意 □部分同意  □同意 □非常同意 |
| 15.你的上级能确保有足够数量的护理人员提供高质量的护理服务  □非常不同意 □不同意 □不太同意 □部分同意  □同意 □非常同意 |
| 16.你的上级在排班时，能考虑员工个人的偏好  □非常不同意 □不同意 □不太同意 □部分同意  □同意 □非常同意 |
| 17.你的上级制定排班表时，能兼顾工作量、患者需求及护理工作的连贯性  □非常不同意 □不同意 □不太同意 □部分同意  □同意 □非常同意 |
| 18.你的上级能不断更新自己循证护理和领导力实践方面的知识  □非常不同意 □不同意 □不太同意 □部分同意  □同意 □非常同意 |
| 19.你的上级能与员工进行有效沟通  □非常不同意 □不同意 □不太同意 □部分同意  □同意 □非常同意 |
| 20.你的上级鼓励员工自由表达意见  □非常不同意 □不同意 □不太同意 □部分同意  □同意 □非常同意 |
| 21.你的上级帮助并促进员工之间建立合作关系  □非常不同意 □不同意 □不太同意 □部分同意  □同意 □非常同意 |
| 22.你的上级会努力营造员工之间相互尊重的文化氛围  □非常不同意 □不同意 □不太同意 □部分同意  □同意 □非常同意 |
| 23.你的上级支持并促进员工培养有效的沟通技巧  □非常不同意 □不同意 □不太同意 □部分同意  □同意 □非常同意 |
| 24.你的上级会积极处理员工之间的冲突，以培养积极的组织文化  □非常不同意 □不同意 □不太同意 □部分同意  □同意 □非常同意 |
| 25.你的上级鼓励护理人员为了患者安全，按照标准执行护理操作  □非常不同意 □不同意 □不太同意 □部分同意  □同意 □非常同意 |
| 26.你的上级能通过人际关系获取医院政策和运营系统的相关信息  □非常不同意 □不同意 □不太同意 □部分同意  □同意 □非常同意 |
| 27.你的上级能够制定与医院整体目标相一致的护理战略计划  □非常不同意 □不同意 □不太同意 □部分同意  □同意 □非常同意 |
| 28.你的上级会积极参与医院内外的管理，例如参与相关政策制定部门和临床实践委员会的工作等  □非常不同意 □不同意 □不太同意 □部分同意  □同意 □非常同意 |
| 29.你的上级致力于构建和管理一套系统，这套系统可以促进护理有效决策和提高护理服务质量  □非常不同意 □不同意 □不太同意 □部分同意  □同意 □非常同意 |
| 30. 你的上级能持续参与制定和实施行动计划和项目，以促进护理质量和服务提升  □非常不同意 □不同意 □不太同意 □部分同意  □同意 □非常同意 |

**Supplementary Table S4.** Demographic Characteristics of Psychometric Validation Participants (n = 662).

| **Variable** | **n** | **%** |
| --- | --- | --- |
| **Age (years)** |  |  |
| 20 ~ 29 | 270 | 40.8 |
| 30 ~ 39 | 222 | 33.5 |
| ≥ 40 | 170 | 25.7 |
| **Gender** |  |  |
| Male | 63 | 9.5 |
| Female | 599 | 90.5 |
| **Educational level** |  |  |
| Junior college | 304 | 45.9 |
| Bachelor's degree or above | 358 | 54.1 |
| **Department** |  |  |
| Medical | 296 | 44.7 |
| Surgical | 255 | 38.5 |
| Intensive Care Unit (ICU) | 61 | 9.2 |
| Others* | 50 | 7.6 |
| **Shift schedule** |  |  |
| Day-evening-night | 348 | 52.6 |
| Day-evening | 165 | 24.9 |
| Fixed night | 98 | 14.8 |
| Fixed day (9 am-5 pm) | 51 | 7.7 |
| **Experience (years), M (SD)** | 2.66 (1.43) |  |
| **Tenure in current position, M (SD)** | 2.45 (1.41) |  |

**Note:** *Others include gynaecology, obstetrics, emergency, and operating room. M (SD), Mean (Standard Deviation)

**Supplementary Table S5.** Item-level descriptive statistics of the CV-INLS (n = 662).

| **Item** | **Mean ± SD** | **Skewness** | **Kurtosis** | **Floor effect (%)** | **Ceiling effect (%)** |
| --- | --- | --- | --- | --- | --- |
| 1 | 4.13 ± 1.52 | -0.66 | -0.63 | 8.16 | 17.82 |
| 2 | 4.09 ± 1.52 | -0.63 | -0.71 | 8.01 | 16.01 |
| 3 | 4.07 ± 1.51 | -0.64 | -0.72 | 8.01 | 14.95 |
| 4 | 4.01 ± 1.55 | -0.63 | -0.81 | 9.82 | 13.75 |
| 5 | 4.16 ± 1.47 | -0.69 | -0.53 | 6.95 | 16.92 |
| 6 | 4.15 ± 1.51 | -0.71 | -0.61 | 7.85 | 16.47 |
| 7 | 3.99 ± 1.55 | -0.60 | -0.82 | 9.67 | 13.60 |
| 8 | 4.09 ± 1.52 | -0.64 | -0.70 | 8.16 | 16.31 |
| 9 | 4.08 ± 1.52 | -0.64 | -0.68 | 8.46 | 16.16 |
| 10 | 4.09 ± 1.51 | -0.68 | -0.64 | 8.46 | 15.26 |
| 11 | 4.11 ± 1.50 | -0.68 | -0.61 | 7.85 | 15.71 |
| 12 | 4.08 ± 1.53 | -0.69 | -0.66 | 9.06 | 15.26 |
| 13 | 4.02 ± 1.50 | -0.61 | -0.72 | 8.16 | 13.44 |
| 14 | 4.06 ± 1.50 | -0.66 | -0.69 | 7.85 | 13.75 |
| 15 | 4.11 ± 1.55 | -0.70 | -0.67 | 9.37 | 16.92 |
| 16 | 4.12 ± 1.55 | -0.69 | -0.68 | 9.06 | 17.07 |
| 17 | 4.09 ± 1.56 | -0.70 | -0.68 | 9.82 | 15.86 |
| 18 | 4.08 ± 1.51 | -0.68 | -0.69 | 8.01 | 14.05 |
| 19 | 4.12 ± 1.51 | -0.75 | -0.51 | 9.21 | 15.41 |
| 20 | 4.16 ± 1.51 | -0.75 | -0.55 | 8.16 | 16.16 |
| 21 | 4.12 ± 1.53 | -0.70 | -0.63 | 8.61 | 16.47 |
| 22 | 4.17 ± 1.51 | -0.73 | -0.60 | 7.55 | 16.92 |
| 23 | 4.08 ± 1.49 | -0.71 | -0.56 | 8.76 | 13.75 |
| 24 | 4.16 ± 1.47 | -0.76 | -0.42 | 7.70 | 15.26 |
| 25 | 4.18 ± 1.53 | -0.76 | -0.51 | 8.76 | 17.82 |
| 26 | 4.15 ± 1.43 | -0.74 | -0.44 | 6.19 | 13.60 |
| 27 | 4.15 ± 1.46 | -0.74 | -0.51 | 6.95 | 14.35 |
| 28 | 4.07 ± 1.47 | -0.67 | -0.61 | 7.40 | 12.99 |
| 29 | 4.09 ± 1.52 | -0.67 | -0.68 | 8.16 | 15.41 |
| 30 | 4.16 ± 1.50 | -0.75 | -0.48 | 8.01 | 16.47 |

**Note:** SD, Standard Deviation

**Supplementary Table S6.** Descriptive statistics of the CV-INLS (n = 662).

| **Dimensions** | **No.** | **Mean ± SD** | **Skewness** | **Kurtosis** | **Floor effect**  **(%)** | **Ceiling effect**  **(%)** |
| --- | --- | --- | --- | --- | --- | --- |
| 1.Personal Leadership Qualities | 9 | 4.09 ± 1.22 | -0.79 | -0.36 | 18 (2.72) | 19 (2.87) |
| 2.Personal Leadership Performance | 9 | 4.08 ± 1.22 | -0.77 | -0.35 | 19 (2.87) | 21 (3.17) |
| 3.Personal Influence on Organizational Culture | 7 | 4.14 ± 1.21 | -0.87 | -0.18 | 19 (2.87) | 17 (2.57) |
| 4.Personal Influence on Organizational Excellence | 5 | 4.12 ± 1.16 | -0.86 | -0.27 | 13 (1.96) | 6 (0.91) |
| Total | 30 | 4.10 ± 1.06 | -0.77 | -0.31 | 7 (1.06) | 2 (0.30) |

**Supplementary Table S7.** Content Validity of the CV-INLS.

| **Item** | **No. of Experts Rating 3 or 4** | **I‑CVI** | **S‑CVI** |
| --- | --- | --- | --- |
| 1 | 14 | 1.000 | 0.993 |
| 2 | 14 | 1.000 |  |
| 3 | 14 | 1.000 |  |
| 4 | 14 | 1.000 |  |
| 5 | 14 | 1.000 |  |
| 6 | 14 | 1.000 |  |
| 7 | 14 | 1.000 |  |
| 8 | 14 | 1.000 |  |
| 9 | 13 | 0.929 |  |
| 10 | 14 | 1.000 |  |
| 11 | 14 | 1.000 |  |
| 12 | 13 | 0.929 |  |
| 13 | 13 | 0.929 |  |
| 14 | 14 | 1.000 |  |
| 15 | 14 | 1.000 |  |
| 16 | 14 | 1.000 |  |
| 17 | 14 | 1.000 |  |
| 18 | 14 | 1.000 |  |
| 19 | 14 | 1.000 |  |
| 20 | 14 | 1.000 |  |
| 21 | 14 | 1.000 |  |
| 22 | 14 | 1.000 |  |
| 23 | 14 | 1.000 |  |
| 24 | 14 | 1.000 |  |
| 25 | 14 | 1.000 |  |
| 26 | 14 | 1.000 |  |
| 27 | 14 | 1.000 |  |
| 28 | 14 | 1.000 |  |
| 29 | 14 | 1.000 |  |
| 30 | 14 | 1.000 |  |

**Note:** I‑CVI = Item‑level Content Validity Index; S‑CVI = Scale‑level Content Validity Index (overall = 0.993).

**Supplementary Table S8.** Factor Loadings of the CV-INLS Items (N=662).

| **Item** | **Factor 1** | **Factor 2** | **Factor 3** | **Factor 4** |
| --- | --- | --- | --- | --- |
| A1 | 0.817 |  |  |  |
| A2 | 0.826 |  |  |  |
| A3 | 0.878 |  |  |  |
| A4 | 0.825 |  |  |  |
| A5 | 0.887 |  |  |  |
| A6 | 0.823 |  |  |  |
| A7 | 0.860 |  |  |  |
| A8 | 0.808 |  |  |  |
| A9 | 0.857 |  |  |  |
| B10 |  | 0.801 |  |  |
| B11 |  | 0.873 |  |  |
| B12 |  | 0.834 |  |  |
| B13 |  | 0.864 |  |  |
| B14 |  | 0.851 |  |  |
| B15 |  | 0.827 |  |  |
| B16 |  | 0.766 |  |  |
| B17 |  | 0.790 |  |  |
| B18 |  | 0.836 |  |  |
| C19 |  |  | 0.774 |  |
| C20 |  |  | 0.837 |  |
| C21 |  |  | 0.790 |  |
| C22 |  |  | 0.742 |  |
| C23 |  |  | 0.822 |  |
| C24 |  |  | 0.840 |  |
| C25 |  |  | 0.827 |  |
| D26 |  |  |  | 0.731 |
| D27 |  |  |  | 0.683 |
| D28 |  |  |  | 0.691 |
| D29 |  |  |  | 0.693 |
| D30 |  |  |  | 0.681 |
| Eigenvalue | 11.718 | 4.614 | 3.457 | 1.680 |
| Percentage of variance explained | 38.021 | 14.288 | 10.509 | 4.149 |
| Total proportion of variance explained by the factor model | | | | 66.967 |

**Note:** Extraction method: principal axis factoring. Rotation method: oblique rotation with Kaiser normalization (converged in 6 iterations). A1–A9 represent Personal Leadership Qualities. B10–B18 represent Personal Leadership Performance. C19–C25 represent Personal Influence on Organizational Culture. D26–D30 represent Personal Influence on Organizational Excellence.

**Supplementary Table S9.** Factor Correlation Matrix from Exploratory Factor Analysis.

| **Factor** | **1** | **2** | **3** | **4** |
| --- | --- | --- | --- | --- |
| 1.Personal Leadership Qualities | 1.000 | 0.212 | -0.462 | 0.364 |
| 2.Personal Leadership Performance | 0.212 | 1.000 | -0.378 | 0.548 |
| 3.Personal Influence on Organizational Culture | -0.462 | -0.378 | 1.000 | -0.350 |
| 4.Personal Influence on Organizational Excellence | 0.364 | 0.548 | -0.350 | 1.000 |

**Note:** Extraction Method: Principal Axis Factoring. Rotation Method: Oblimin with Kaiser Normalization.

**Supplementary Table S10.** Results of the convergent and discriminant validity of the CV-INLS.

| **Factors** | **CR** | **AVE** | **1** | **2** | **3** | **4** |
| --- | --- | --- | --- | --- | --- | --- |
| 1. Personal Leadership Qualities | 0.968 | 0.771 | 0.878 |  |  |  |
| 1. Personal Leadership Performance | 0.964 | 0.749 | 0.421 | 0.866 |  |  |
| 1. Personal Influence on Organizational Culture | 0.946 | 0.714 | 0.344 | 0.158 | 0.845 |  |
| 1. Personal Influence on Organizational Excellence | 0.830 | 0.494 | 0.373 | 0.338 | 0.546 | 0.703 |

**Note:** CR, composite reliability. AVE, average variance extracted.

**Supplementary Table S11.** Item and Dimension Mean Scores of the CV-INLS (n = 1, 023).

| Dimension | Item | Item Score  (x̄±SD) | Dimension Score  (x̄±SD) | Theoretical Range | Min | Max |
| --- | --- | --- | --- | --- | --- | --- |
| Personal Leadership Qualities | 1 | 4.06 ± 1.53 | 36.35 ± 11.41 | 1 ~ 6 | 1 | 6 |
|  | 2 | 4.05 ± 1.53 |  | 1 ~ 6 | 1 | 6 |
|  | 3 | 4.03 ± 1.55 |  | 1 ~ 6 | 1 | 6 |
|  | 4 | 3.97 ± 1.56 |  | 1 ~ 6 | 1 | 6 |
|  | 5 | 4.08 ± 1.48 |  | 1 ~ 6 | 1 | 6 |
|  | 6 | 4.11 ± 1.54 |  | 1 ~ 6 | 1 | 6 |
|  | 7 | 3.95 ± 1.55 |  | 1 ~ 6 | 1 | 6 |
|  | 8 | 4.05 ± 1.56 |  | 1 ~ 6 | 1 | 6 |
|  | 9 | 4.06 ± 1.54 |  | 1 ~ 6 | 1 | 6 |
| Personal  Leadership Performance | 10 | 4.07 ± 1.55 | 36.41 ± 11.44 | 1 ~ 6 | 1 | 6 |
|  | 11 | 4.05 ± 1.53 |  | 1 ~ 6 | 1 | 6 |
|  | 12 | 4.04 ± 1.57 |  | 1 ~ 6 | 1 | 6 |
|  | 13 | 4.00 ± 1.52 |  | 1 ~ 6 | 1 | 6 |
|  | 14 | 4.03 ± 1.51 |  | 1 ~ 6 | 1 | 6 |
|  | 15 | 4.09 ± 1.57 |  | 1 ~ 6 | 1 | 6 |
|  | 16 | 4.03 ± 1.57 |  | 1 ~ 6 | 1 | 6 |
|  | 17 | 4.06 ± 1.57 |  | 1 ~ 6 | 1 | 6 |
|  | 18 | 4.05 ± 1.53 |  | 1 ~ 6 | 1 | 6 |
| Personal  Influence on Organizational Culture | 19 | 4.08 ± 1.53 | 28.84 ± 8.77 | 1 ~ 6 | 1 | 6 |
|  | 20 | 4.11 ± 1.53 |  | 1 ~ 6 | 1 | 6 |
|  | 21 | 4.10 ± 1.54 |  | 1 ~ 6 | 1 | 6 |
|  | 22 | 4.16 ± 1.53 |  | 1 ~ 6 | 1 | 6 |
|  | 23 | 4.06 ± 1.51 |  | 1 ~ 6 | 1 | 6 |
|  | 24 | 4.14 ± 1.48 |  | 1 ~ 6 | 1 | 6 |
|  | 25 | 4.19 ± 1.54 |  | 1 ~ 6 | 1 | 6 |
| Personal Influence on  Organizational Excellence | 26 | 4.13 ± 1.45 | 24.62 ± 7.12 | 1 ~ 6 | 1 | 6 |
|  | 27 | 4.13 ± 1.46 |  | 1 ~ 6 | 1 | 6 |
|  | 28 | 4.05 ± 1.48 |  | 1 ~ 6 | 1 | 6 |
|  | 29 | 4.06 ± 1.52 |  | 1 ~ 6 | 1 | 6 |
|  | 30 | 4.16 ± 1.49 |  | 1 ~ 6 | 1 | 6 |
| Total score |  | 118.04 ± 29.69 | | 30 ~ 180 | 30 | 180 |

|  |
| --- |

**Note:** x̄, mean. SD, standard deviation. The total score was calculated by summing the scores of all 30 items (range: 30–180).

**Supplementary Table S12.** Differences in perceived integral nursing leadership among nurses with different characteristics (n = 1,023)

| **Variables** | **Mean ± SD** | **t / F** | **P** |
| --- | --- | --- | --- |
| **Age** |  |  |  |
| 20 ~ 29 | 102.04 ± 25.14 | 206.832 | 0.000 |
| 30 ~ 39 | 121.93 ± 24.76 |  |  |
| ≥40 | 142.16 ± 25.38 |  |  |
| **Gender** |  |  |  |
| Male | 110.85 ± 28.78 | 6.026 | 0.014 |
| Female | 118.76 ± 29.71 |  |  |
| **Educational Level** |  |  |  |
| Junior college | 114.84 ± 29.97 | 9.100 | 0.003 |
| Bachelor's degree or above | 120.47 ± 29.28 |  |  |
| **Department** |  |  |  |
| Medical | 121.43 ± 29.36 | 8.310 | 0.000 |
| Surgical | 118.84 ± 28.60 |  |  |
| Intensive Care Unit (ICU) | 110.32 ± 32.57 |  |  |
| Other | 108.31 ± 29.64 |  |  |
| **Shift Schedule** |  |  |  |
| Day-evening-night | 117.36 ± 28.08 | 0.347 | 0.792 |
| Day-evening | 118.24 ± 30.45 |  |  |
| Fixed night | 118.64 ± 32.49 |  |  |
| Fixed day (9 am-5 pm) | 120.57 ± 32.96 |  |  |
| **Experience (years)** |  |  |  |
| 1 ~ 9 | 103.25 ± 25.25 | 208.484 | 0.000 |
| 10 ~ 19 | 125.60 ± 25.24 |  |  |
| >20 | 145.19 ± 24.06 |  |  |
| **Tenure in current position (years)** |  |  |  |
| 1 ~ 9 | 104.42 ± 25.45 | 202.23 | 0.000 |
| 9 ~ 16 | 127.45 ± 24.43 |  |  |
| >16 | 141.83 ± 29.69 |  |  |
